# Expanding Paramedicine in the Community (EPIC): study protocol for a randomized controlled trial

**DOI:** 10.1186/1745-6215-15-473

**Published:** 2014-12-02

**Authors:** Ian R Drennan, Katie N Dainty, Paul Hoogeveen, Clare L Atzema, Norm Barrette, Gillian Hawker, Jeffrey S Hoch, Wanrudee Isaranuwatchai, Jane Philpott, Chris Spearen, Walter Tavares, Linda Turner, Melissa Farrell, Tom Filosa, Jennifer Kane, Alex Kiss, Laurie J Morrison

**Affiliations:** Rescu, Li Ka Shing Knowledge Institute, St. Michael’s Hospital, 30 Bond St, Toronto, ON M5B 1W8 Canada; Institute of Medical Science, Faculty of Medicine, University of Toronto, Toronto, ON Canada; York Region Emergency Medical Services, Newmarket, ON Canada; Sunnybrook Center for Prehospital Medicine, University of Toronto, Toronto, ON Canada; Division of Emergency Medicine, Department of Medicine, University of Toronto, Toronto, ON Canada; Institute for Clinical Evaluative Sciences, Toronto, ON Canada; Sunnybrook Health Sciences Center, Toronto, ON Canada; Women’s College Hospital, Toronto, ON Canada; Department of Medicine, University of Toronto, Toronto, ON Canada; Institute of Health Policy, Management and Evaluation, University of Toronto, Toronto, ON Canada; Center for Excellence in Economic Analysis Research, Li Ka Shing Knowledge Institute, St. Michael’s Hospital, Toronto, ON Canada; Department of Family Medicine, Markham Stouffville Hospital, Markham, ON Canada; Health For All Family Health Team, Markham, ON Canada; Department of Family and Community Medicine, University of Toronto, Toronto, Canada; University of Toronto Wilson Center for Health Professions Education, Toronto, ON Canada; Department of Community and Health Studies, Paramedic Program, Centennial College, Toronto, ON Canada; Paramedic Association of Canada, Ottawa, ON Canada; Primary Health Care Program, Ministry of Health and Long-Term Care, Toronto, Ontario Canada; Markham Family Health Team, Markham, ON Canada

**Keywords:** Randomized controlled trial, Community health services, Primary health care, Allied health personnel

## Abstract

**Background:**

The incidence of chronic diseases, including diabetes mellitus (DM), heart failure (HF) and chronic obstructive pulmonary disease (COPD) is on the rise. The existing health care system must evolve to meet the growing needs of patients with these chronic diseases and reduce the strain on both acute care and hospital-based health care resources. Paramedics are an allied health care resource consisting of highly-trained practitioners who are comfortable working independently and in collaboration with other resources in the out-of-hospital setting. Expanding the paramedic’s scope of practice to include community-based care may decrease the utilization of acute care and hospital-based health care resources by patients with chronic disease.

**Methods/Design:**

This will be a pragmatic, randomized controlled trial comparing a community paramedic intervention to standard of care for patients with one of three chronic diseases. The objective of the trial is to determine whether community paramedics conducting regular home visits, including health assessments and evidence-based treatments, in partnership with primary care physicians and other community based resources, will decrease the rate of hospitalization and emergency department use for patients with DM, HF and COPD. The primary outcome measure will be the rate of hospitalization at one year. Secondary outcomes will include measures of health system utilization, overall health status, and cost-effectiveness of the intervention over the same time period. Outcome measures will be assessed using both Poisson regression and negative binomial regression analyses to assess the primary outcome.

**Discussion:**

The results of this study will be used to inform decisions around the implementation of community paramedic programs. If successful in preventing hospitalizations, it has the ability to be scaled up to other regions, both nationally and internationally. The methods described in this paper will serve as a basis for future work related to this study.

**Trial registration:**

ClinicalTrials.gov: NCT02034045. Date: 9 January 2014.

**Electronic supplementary material:**

The online version of this article (doi:10.1186/1745-6215-15-473) contains supplementary material, which is available to authorized users.

## Background

Patients with chronic diseases have high rates of health care utilization and are currently costing the health care system billions of dollars each year [1–3]. In order to effectively care for the increasing number of individuals living with chronic disease, there has emerged a need for novel community-based health initiatives. Individuals with diabetes mellitus (DM) are over 3 times more likely to be hospitalized with cardiovascular disease than individuals without diabetes, 12 times more likely to be hospitalized with end-stage renal disease, and almost 20 times more likely to be hospitalized with non-traumatic lower limb amputations [4]. There are 5.5 million people in the United States and Canada living with heart failure (HF) with 850,000 new patients diagnosed annually [5, 6] and more than 900,000 hospitalizations a year are attributable to HF [6–8]. Chronic obstructive pulmonary disease (COPD) is one of the fastest growing chronic diseases; it is the third leading cause of death in the United States [9] and fourth leading cause of death in Canada, [10] and has an average hospitalization of 10 days for an exacerbation with an associated cost of $10,000 [11]. The existing health care system, which primarily revolves around hospital-based care and primary care providers, must evolve to incorporate new strategies of delivering care to better meet the needs of this growing population, resulting in improved patient care and more efficient resource utilization.

To this end, the addition of community paramedics may enrich the system that currently manages these patients. Paramedics are a highly-trained group of prehospital practitioners certified in both basic and advanced life support care who work under the medical delegation of base hospital physicians. Paramedics are mobile, community-based practitioners accessible 24 hours a day, who are efficient and effective in managing emergencies and providing comprehensive care in the out-of-hospital environment [12]. Paramedics are the first, and sometimes only, point of contact for many in the community for disease-related exacerbations/symptomatology. They are able to identify subtle signs of potentially life-threatening issues, and provide comprehensive care as per medical directives or reach out to on-call physicians to address issues at the bedside before they become emergencies.

The integration of community paramedics into health care models has attracted international attention. Studies in the United Kingdom, Australia and the United States have shown that community paramedic implementation is feasible, safe and effective while at the same time improving satisfaction of patients and practitioners and minimizing health care costs [13]. Despite this, there has been minimal adoption of community paramedic programs in Canada. While the evidence supporting community paramedicine is promising a comprehensive systematic review identified only a single randomized controlled trial (RCT) from the United Kingdom that evaluated the efficacy of community paramedics [14]. By combining in-home visits by community paramedics with physician oversight and integration with existing community resources, the Expanding Paramedicine in the Community (EPIC) study will provide an innovative chronic care model in order to maximize patient care by allotting available resources in a tiered delivery system.

We propose to perform a randomized controlled trial to answer the following question: does expanding paramedic scope of practice to include chronic disease management, characterized by home visits to facilitate the assessment and treatment of patients under the medical delegation of the primary care physician, reduce the rate of acute care hospitalization?

## Methods/Design

This stratified, randomized controlled trial will examine the effectiveness of a community paramedicine intervention in reducing hospital admission among family health team patients diagnosed with either COPD, HF or DM. We consider our trial to be pragmatic [15] for the following reasons: (1) the patient populations will be broad, targeting patients at high-risk, with few exclusion criteria; (2) the intervention will be implemented in a ‘usual’ practice setting; (3) the intervention will be flexible, and may be adapted according to identified needs over the study period; (4) the outcomes are patient-centered; and (5) the trial results will be of interest to decision-makers [15, 16]. The trial has received research ethics board approval from St. Michael’s Hospital and Markham Stouffville Hospital (Research Ethics Board # 13-086). See Additional files 1 and 2.

### Setting

The regional municipality of York is a geographically diverse region in southern Ontario with a mix of rural and suburban centers and a population of just over 1 million residents. York Region Emergency Medical Services (EMS) is the sole provider of 911 medical services in York Region with both primary care paramedics and advanced care paramedics who are certified through the Ministry of Health and Long-Term Care (MOHLTC). They operate under the medical oversight of physicians from a single base hospital (Central East Prehospital Care Program) and are employed to provide medical care and transportation services within the region.

Patients for this study will be recruited from the Markham Family Health Team and the Health For All Family Health Team, the two largest family health teams in York Region, each with over 5,000 subscribing adult patients.

### Patient population

Patients of the participating family health teams diagnosed with either HF, COPD or DM will be screened for potential study eligibility by their primary care physician by applying search filters to the patients’ electronic medical records (EMR). Eligibility criteria are listed in Table 1. Patients will be deemed to be high-risk at the discretion of the primary care physician, based on (i) prior patterns of health care use, (ii) test results, (iii) compliance and (iv) clinical *gestalt*. Enrolled patients will be confirmed to be high-risk for admission by determining their rates of hospital admission over the 3 years prior to study enrollment, through linkage with the Institute for Clinical Evaluative Sciences (ICES) databases. Rates of admission will then be compared to previous literature to determine patients who are high-risk.

**Table 1:** **Study eligibility criteria**

Eligibility criteria	Ineligibility criteria
Patients are eligible if:	Patients are ineligible if:
• They are residents of the region of York	• They are residents of long-term care facilities
• They are 18 years of age or older	• They have cognitive impairment, uncontrolled psychiatric disease or language barriers that would make it difficult to understand the consent and communicate with the paramedic during the scheduled visits, unless the individual with power of attorney for personal care consented and agreed to be at each visit
• They have been diagnosed (at any point in time prior to enrollment) with, and currently receiving treatment for COPD, HF, or DM	
• They are identified by the Family Health Care Team as being at high-risk for hospital admission	

### Recruitment and randomization

**Figure 1:**
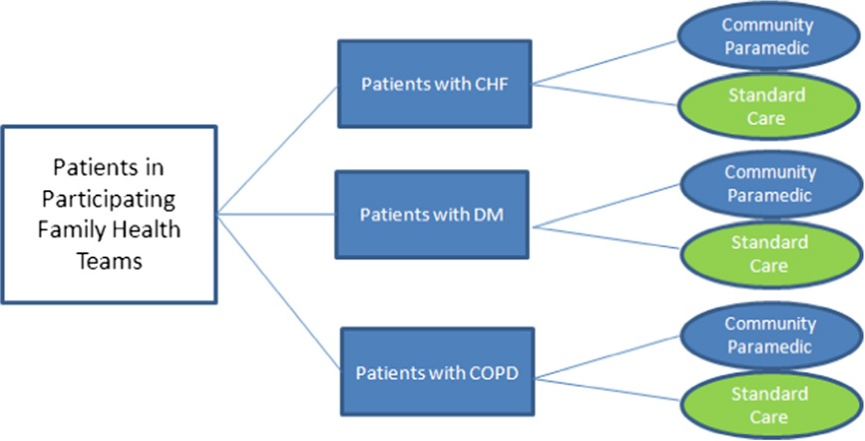
**Patient block stratified randomization procedure.**

Eligible patients will be contacted by their family physician and given multiple opportunities to provide their consent to participate. Enrollment will employ a modified Dillman method [17] with prepaid postage response cards. All patients will be consented using exactly the same process prior to randomization. See Additional files 3 and 4.A block stratified randomization procedure will be used to randomize eligible, consenting patients to treatment or control groups (1:1 ratio). Randomization will occur using a computer generated randomized number sequence allocated by block based on disease, using variable block sizes to avoid substantial imbalances in the number of patients assigned to each group (Figure 1) and to ensure the three disease groups are represented in the intervention or control (usual care) group. Stratification will balance differences in disease characteristics, which may influence the primary outcomes. Should patients have multiple diagnoses, patients with HF will be categorized HF regardless of the other diagnoses and patients with both COPD and DM will be categorized as COPD. Categorization is based on the prevalence of disease previously noted in a subset of patients in which the number of patients diagnosed as having HF was markedly reduced. A preliminary feasibility sample of cases from within the two family health care teams identified 548 patients: 74 COPD patients, 417 DM patients, and 31 HF patients. One patient had all 3 conditions, 17 had diabetes and HF, 1 had HF and COPD and finally 7 patients had both diabetes and COPD. Informed consent will be obtained from all patients prior to enrollment in the EPIC study. Patient recruitment will occur over a 1-year period. Based upon the preliminary feasibility sample and the sample size calculation required to detect a 15% difference in our primary outcome, this will be sufficient time to enroll the required number of patients.

### Planned treatment groups

The intervention will consist of an initial visit and 3 follow-up visits at 3-month intervals by a paramedic who has received additional training in chronic disease management. The specially designed training consists of a 6-week in-class education program designed and provided by Centennial College in Toronto, Ontario, Canada, expanding the paramedic’s scope of care for this patient population and non-emergency practice. This curriculum will include didactic, clinical (that is specialized disease specific clinics), skills and immersive simulation/case-based instruction. The didactic portion will include the following courses: Health Assessment, Diagnostics and Care (theory and hands-on practice), Pathophysiology, Pharmacology and Health Teaching and Promotion. Core competencies target foundational knowledge, communication, assessment and diagnostics, therapeutics including pharmacology, integration, and health promotion. Clinical placements under the direct supervision of specialists or physicians along with simulation-based practice will occur concurrently to flexibly engage students in relevant cases to promote application of concepts, skill development, clinical reasoning and transfer. A total of seven community paramedics have been trained. This will be a sufficient number of paramedics to carry out the planned patient visits for the intended sample size, as well as additional time set aside for visits initiated outside of the planned visits. This is based on a total number of 168 hours per week (7 am to 7 pm, 5 days a week) during which 1 of 2 community paramedics are available for appointments. The intervention will ensure a standardized baseline number of visits by the community paramedics and will occur in addition to routine usual care and any additional health care visits prompted by the patient, the paramedic or the family health care team. The initial visit and each follow-up visit by the community paramedic will include a medical history and physical examination based on disease-specific elements recorded on an electronic assessment tool that is e-linked to the patient care record for the entire family health care team. Patients will receive disease-specific education and counseling in accordance with the community paramedic medical directives. If necessary, the community paramedic may initiate treatment in the home, based on disease-specific evidence-based medical directives and/or may initiate telephone contact with the primary health care physician in accordance with their medical directive. If treatment is initiated by community paramedics, patients are expected to follow up with their family physician within 72 hours. Additional primary care physician and/or community paramedic follow-up appointments may be scheduled by the paramedic as required. Community Care Access Center (CCAC) and other community resources will also be notified at the discretion of the primary care physicians or other family health care team members. The patient will be able to notify their family health team or the community paramedic about the need for a subsequent assessment based on any change in their condition.

Patients randomized to the control group will continue to receive usual care from their family health care team. Usual care includes physician assessment and treatment and periodic augmentation of care in the community (CCAC case manager or nurse practitioner) at the discretion of the treating physician.

### Considerations to reduce bias

Due to the intended treatment plan it is not possible to blind paramedics, clinicians or research personnel to treatment assignment. To help minimize selection bias, randomization will occur via a computer-generated random number formulation. Detection bias will be minimized through linkage to copies of province-wide administrative datasets held at ICES. Ascertainment bias will be controlled by using objective clinical outcomes that are not under the control of the paramedic or the family health care team (for example, hospital admission, length of stay, death). Attrition bias is avoided as we are using health outcomes that are routinely measured and housed as administrative data points, and using a primary intention-to-treat (ITT) analysis will minimize the potential bias due to patient withdrawal, dropout or study protocol violations. Contamination bias will be reduced as eligible patients from the same family will be assigned to the first family member’s randomly assigned treatment group. Missing data will be minimized through point of care logic and error checks, built-in data management reports run weekly and timely oversight by study staff; however, any remaining missing data will be dealt with using multiple imputation. The statistical measures of our data management system are published [18]; there is > 90% agreement in the variables of interest common to all cases with moderate or excellent kappa (0.65 to 0.87).

### Proposed primary and secondary outcome measures

The primary outcome of this trial is the number of hospitalizations per patient after one year of study enrollment. Secondary outcomes will be measures of health system utilization at 1-year (reported as all cause and disease specific) and will include the following:

1. Calls to 911 (regardless of whether patient was transported to hospital)
2. Visits to the participating family health team clinics and any after-hours clinics
3. Length of stay in hospital if admitted
4. Mortality
5. Overall health status assessment using the (Euroqol-5D 3L) EQ-5D-3L measured at baseline and one year after study enrollment [19, 20]
6. Measures of intervention compliance and safety (that is completed assessments and visits and protocol violations identified by physician review of EMR)
7. Cost-effectiveness analysis for this model of care

The family health team electronic patient record data will be linked via the health card number to province-wide administrative health datasets held at the ICES, a section 45 Prescribed Entity under Ontario’s Personal Health Information Protection Act. Health services data at ICES represent ‘transactions’ of health care utilization, such as inpatient/outpatient hospital utilization, emergency room and other ambulatory clinic visits, drug claims for patients 65 and older, claims for physician services in any setting, and others. Linkage to the ICES administrative databases requires use of the Ontario health card number for each subject; following linkage, only the unique ICES encrypted identifier will remain on the files used for analysis. Linkage will allow us to obtain a more comprehensive view of specific health system utilization than could be achieved with unlinked data. Many disease-specific codes have been validated in ICES’ databases [21–23].

Patients will be asked about health-related quality of life using the EQ-5D-3 L, [20] a validated measure of quality-adjusted life years (QALY). QALY, a preference-based utility measure, incorporates both length of life and quality of life into a single measure. To estimate QALYs, we will convert EQ-5D data collected in the study to utility score using a validated algorithm [19] so no additional data collection will be required. Health-related quality of life assessments will be performed at baseline and 1-year after study enrollment. Assessments will be performed by the community paramedics for patients enrolled in the intervention cohort and by the patients’ primary care physicians in the control cohort.

### Sample size

The proposed sample size is based on yearly hospitalization rates in Ontario of 296/100,000 for HF, [24] 632/100,000 for COPD [25] and 67/100,000 for DM [26] and 1-year mortality rates of 10% for HF, [27] 4.4% for COPD [28] and 8.2% for DM [29]. A Poisson regression with a sample of 695 (348 per group) achieves 80% power at a 0.05 significance level to detect a response rate ratio of at least 0.85 between groups (intervention and control) given a baseline rate of 1.85 and an assumed phi (over-dispersion parameter) of 1.0. The sample size was adjusted since a multiple regression of the covariate of interest on the other covariates in the Poisson regression is expected to have an R-squared of 0.05. We believe this to be sufficient power to detect a clinically relevant improvement of 15% in hospital admission rates. The proposed sample size calculation will be confirmed by using the baseline rates of admission as determined through ICES for patients enrolled in the EPIC study.

### Data collection and management

#### I. Confidentiality and security

Data regarding patient assessment and treatment is currently collected in the family health team’s EMR and is remotely accessed by health care providers (physicians, social workers, dieticians, physician assistants and nurse practitioners). CCAC and the community paramedics will be joining the patients’ circle of care and will sign confidentiality agreements to be provided with EMR access.

#### II. Data abstraction

Trained data abstractors will enter data from patients’ hospital visits into the EMR. They will be trained by the study staff and complete all the necessary privacy and confidentiality paperwork prior to beginning data abstraction. Patient data will be collected at each encounter with the community paramedic, both scheduled and unscheduled. Data from the paramedic reports and family health team EMRs will be abstracted and manually entered into the database housed at St. Michael’s Hospital on a continual basis throughout the study in real time. Outcome data (that is emergency department (ED) visits, hospital admissions, calls to 911) will be collected at study completion for each patient.

#### III. Data entry and data storage

All study data and clinical variables collected in the EMR will be stored on St. Michael’s Hospital research data servers. This server site is in compliance with Personal Health Information Privacy Act (PHIPA) guidelines. This password protected database has restricted access in compliance with the privacy and ethical practices of St. Michael’s Hospital. Access to the room, which contains the research file server, is restricted to designated persons who are employed by St. Michael’s Hospital. The database is stored electronically on this file server that is protected by a firewall, and making it inaccessible externally. Safeguards are in place to protect personal health information against loss, theft, as well as unauthorized access, disclosure, copying, use, or modification. The nature of the safeguards will vary depending on the format of the information, and the method of storage. Only co-investigators will have access to the final trial dataset on a request basis via the primary investigator.

#### IV. Privacy considerations for study intervention

Protecting the privacy of the subjects whose health information is obtained and housed by our research program is a mandatory component of conducting ethical clinical studies and is of utmost importance. This commitment is demonstrated through the adherence to and the compliance with Canada’s federal privacy law, the Personal Information Protection and Electronic Documents Act 2000 (PIPEDA - current to 24 March 2011), the Ontario privacy legislation, and PHIPA 2004 (last amendment: 2010).

### Data analysis

Clinical trials involving community-based medicine and prehospital providers are conducted in relatively uncontrolled environments. These settings can be unpredictable and pose many unique operational and analytical challenges for even the most rigorously designed clinical trials such as loss of patient follow-up and problems with data capture. To accommodate such challenges in a manner that maintains applicability to usual clinical practice yet ensures scientific integrity, study outcomes will be evaluated in two populations: an ITT or ‘safety population’ as our primary analysis and a modified intention-to-treat (MITT) or ‘efficacy population’ as a sensitivity analysis.

### Outcome analyses

The MITT or efficacy population will consist of all eligible randomized patients who met all eligibility criteria and who received the full study protocol (4 scheduled paramedic visits over a 1-year period or patients randomized to the control group who remained in the study for the full 1-year period). Furthermore, this trial is intended to target patients with chronic disease who are at high-risk for admission to hospital. However, initial patient enrollment into the study was based upon family physician estimation of patient risk; therefore there is a possibility that some patients enrolled in the study do not represent a high-risk group for hospital admission. Patients enrolled in the trial will have their risk of admission assessed based on their actual rates of admission over the previous 3 years. This information will be gathered through linkage with the ICES databases. Cut-off values for low, medium and high-risk categories will be calculated based on a review of the literature and clinical expertise. Only those patients who are determined to be high-risk will be included in the MITT analysis. Conversely, the ITT or effectiveness population includes all patients randomized regardless of their eligibility, adherence to study protocol or calculated risk of admission.

The primary analysis will be done using an ITT analysis. Baseline characteristics of patients in the intervention and control groups within each disease-specific group will be reported using descriptive statistics, mean/SD or median/IQR for continuous variables and count/percent for categorical as appropriate. Tests of significance will be performed using *t*-tests or Wilcoxon rank-sum test for continuous variables and chi-square or Fisher’s exact test (expected counts < 5) for categorical variables as appropriate. Two-sided *P*-value < 0.05 will be used to determine significant differences in baseline characteristics. These analyses are useful not only to assess the comparability of the treatment group but also to describe the sample of subjects who entered the trial. Test of normality for each variable will be determined using the Shapiro-Wilk test *P*-value < 0.05. All tests will be two-sided and carried out at a significance level at 5%.

The primary outcome, number of hospital admissions over 1 year will be compared by group using both Poisson regression and negative binomial regression analyses. The goodness of fit of both models will be evaluated using Akaike’s information criterion (AIC) statistics. If, in spite of randomization, differences in baseline characteristics are found using bivariate analytical strategies, these variables will be included in the regression model. Multiple imputation algorithms will be developed in order to determine values for any missing data points [30].

The secondary outcome measures, which are largely count measures, will be compared between control and intervention groups using similar Poisson regression and negative binomial models. The proportion of patients who die during the study period will be compared for the intervention and control groups within each disease-specific group using chi-square tests as well as a logistic regression model, adjusting for any potential confounding variables as appropriate.

### Economic analysis

Subsequent to the outcome analysis, we will conduct an economic analysis. The objective of the economic analysis will be to compare the relative costs and effects of a community paramedicine intervention with usual care. We will conduct the analysis from the perspective of the MOHLTC using data from the randomized controlled trial and ICES. Since the effectiveness outcomes are hospitalization, ED visits, primary care visits, EMS utilization and medication use, which can be expressed in monetary terms (for example, hospitalization cost), we will convert the outcomes to dollars and will analyze the costs of the two groups. Total cost for each patient includes the cost of intervention and the health care costs incurred within 1-year following randomization (as defined in the outcome measures sections). Based on initial intervention assignment, we will analyze the cost variable as a dependent variable to estimate the difference in expected health care cost with a study group as the primary independent variable in a regression model. Employing regression will allow for the adjustment of potential confounders (for example, patient characteristics).

In theory, an ordinary least squares (OLS) model produces unbiased estimates even if the data are skewed; [31] however, additional estimation methods (for example, generalized linear models [32]) and different uncertainty methods (for example, parametric and non-parametric confidence intervals) will facilitate careful investigation of the impact various assumptions have on our conclusions. In addition, we will conduct a cost-effectiveness analysis using mortality and QALY as the outcome of interest. Methods for censored cost and outcome data will be accommodated in a net benefit regression framework [33, 34].

### Subgroup analyses

We will conduct subgroup analyses to determine whether any of the following factors modify the effect of community paramedic interventions on study outcomes: family health team allocation, socioeconomic status, living alone, immigrant status, age, gender, distance from clinic and home care utilization.

An interim safety analysis will be conducted after 6 months from the last primary assessment of the first 50% enrolled using data from family health team EMRs and paramedic ePCRs. Interim analysis will be conducted to evaluate the intervention for issues relating to patient safety predefined as community paramedic non-compliance with specified protocols and physician interventions post-paramedic assessment.

### Knowledge translation and dissemination of results

There will be a fully integrated knowledge translation (KT) and dissemination approach over the course of the study with involvement from a number of community and government stakeholders. During the evaluation we will have regular project feedback presentations open to all stakeholders (patients, family health teams, paramedics, EMS services, CCAC, MOHLTC) with summaries circulated to all participating organizations. We will have a regular project newsletter to share implementation information and interim findings and organize stakeholder conferences with the intervention team and our MOHLTC partners to keep the lines of communication open regarding the community paramedicine approach.

We will use traditional end-of-grant KT strategies (for example, presentations, publications, press releases, social media strategies including animated shorts, and so on) to share lessons from EPIC as well as to disseminate its results. We will present findings at national and international academic research conferences to a wide range of audiences to ensure that we target the broad range of disciplines involved in this study.

## Discussion

While the evidence supporting community paramedicine is promising, there has yet to be a convincing trial evaluating the impact of community paramedicine on the health outcomes of, and health system utilization costs associated with, chronic disease patients. EPIC employs a chronic care model that integrates with the current health care system, and has the potential to positively impact health outcomes for individuals living with DM, COPD and HF. Chronic care models of care delivery are integrated disease management plans consisting of self-management support, clinical decision support, a unified clinical information system, and organization of health care and community resources [35]. Disease management plans with two or more components of care have been shown to shorten hospital length of stay as well as decrease rates of hospitalizations and ED visits [35]. The most effective disease management plan enables the adjustment of treatment [36] through high-frequency patient contact and case management [37]. Using a regional, community-based strategy, the EPIC study will implement such a disease management plan in order to reduce rates of hospital admission and emergency department visits for those living with at least one of the three most common chronic diseases: DM, HF and COPD.

We believe that the EPIC intervention has the potential to significantly impact the management of chronic health disease, via reduced hospitalizations and ED visits, which in turn reflects quality of care [38]. Our intervention is designed to work within the existing health care system to maximize the quality of care it delivers as well as the use of existing resources. The simplicity of the intervention means that, if proven useful, community paramedicine could easily be scaled up to other EMS services and community health care teams and expanded to cover a broader range of health conditions and populations of individuals.

Paramedics are an under-utilized resource, well suited to provide community-based health care to patients in their homes. They are highly-trained health care practitioners who are mobile in the community and have the infrastructure in place to provide home care services; however, they are limited in some communities to responding to 911 emergency calls. They have a unique skill set, are able to use their surroundings to capture information, identify subtle signs of potentially life-threatening issues, and provide comprehensive care as per medical directives or through conversation with on-call physicians to address issues at the bedside before they become emergencies. Paramedics are ideally suited to provide integrated care above and beyond current community resources to provide care for patients with chronic disease. Given that they work under the medical delegation of base hospital physicians, paramedics have the ability to provide active treatment that other health care providers, such as nurses working in the homecare setting, cannot. In communities with low population density, paramedics have the potential to expand their scope of practice and provide care during predictable low volume time intervals. In addition, current EMS deployment models suggest that on any given day up to 20% of the paramedic workforce is on modified duty and unable to manage the heavy lifting required for emergency call responses [39]. This group could be refocused on community paramedicine booked appointments, while other physically fit colleagues maintain current EMS performance standards [39]. Extending a paramedic’s current knowledge of chronic disease to include routine care and prevention of morbidity and mortality will require a modest investment in education; however, there are anticipated future year cost avoidance benefits. Current projections anticipate the need to increase EMS staff by at least 50% over the next 10 years [39] to address the shift to an aging population; this could be mitigated in part by community paramedicine programs as part of an integrated system.

Despite the many theoretical advantages and the anecdotal evidence there is a lack of quality research evaluating the effects of community paramedicine programs on patient care and safety, health care systems and economic impact. In Nova Scotia, a longitudinal mixed-methods study was undertaken that utilized a nurse practitioner-paramedic-family physician care model [40]. Community paramedics performed diabetic assessments, wound care, congestive heart failure assessments, drew blood for subsequent lab tests, and provided education sessions. Between year 1 (pre-intervention) and year 3 (year following implementation of intervention), there was a 40% decrease in ED visits, 28% decrease in family physician visits and a marked decrease in mean total health costs [40]. A recent systematic review [13] identified only one randomized controlled trial from the United Kingdom that evaluated the effectiveness of community paramedics [14]. Although this study identified benefits to the use of community paramedic practitioners in the UK it was limited by methodological flaws including; quasi-randomization procedures, a significant proportion of patients who did not receive the treatment they were assigned, and poor response to the survey questionnaire [14]. In the interests of economic efficiency, it is imperative that studies be undertaken to properly evaluate the role of community paramedics before these programs undergo widespread implementation. Furthermore, the study will measure the reduction of burden placed on the existing health system, both in time and cost, by delivering quality patient care at the non-acute level.

Like any pragmatic randomized trial implemented in a real-world setting, we anticipate there will be a few challenges in conducting this study. There are some threats to follow-up such as the inability to complete the scheduled paramedic visits and the possibility of missing outcome data. The community paramedics will be responsible for setting appointments and ensuring that the patients have adequate notification. The paramedics will travel to the patient’s home for the appointment, which by design will minimize the risk of missed appointments; however patients could go on extended holidays, move away or select to discontinue in the study, which would affect the ability to complete the intervention. The resulting inability to measure outcomes in these patients will be minimized by using datasets linked by a universal health insurance number documented by the paramedic at the level of the patient as well as several source documents. We have a track record of diligence in data capture and outcome measurement; our current randomized trials of prehospital interventions have < 1% missingness for benchmarked critical variables and loss to follow-up for primary and secondary outcomes [41, 42]. A modest threat of *selection and ascertainment bias* exists regardless of the strategies we have described previously to try to minimize this risk of bias.

If found to be effective, this pragmatic intervention is designed such that it could be implemented in many jurisdictions, which would extend the benefits of the intervention to many more patients living with these chronic diseases. It is expected that our study will be able to provide rigorous answers regarding the utility of the intervention in improving health outcomes for patients with chronic diseases.

## Trial status

In order to test feasibility of the intervention process, as well as ease of patient recruitment, a pilot study of the EPIC protocol has been underway since March 2013. To date, the EPIC team has recruited 22 physicians from 2 family health teams and 6 paramedics from York Region EMS. Each physician has reviewed and signed off on the medical directives and processes have been created and tested to enable online physician consultation during the EPIC visits, as well as physician review of documentation and intervention post visit. A search of the family health team’s EMR dataset using the study inclusion criteria identified an initial list of potential patients, which was then reviewed by the physicians. A preliminary sample of eligible cases identified 548 patients, and we consented and randomized 210 (38.4%) patients over a several week period. Each patient provided informed consent prior to participation in the study.

The community paramedics have undergone a 6-week education program designed and provided by Centennial College, expanding their scope of care for this patient population and non-emergency practice. The paramedics have also successfully completed EMR training, data entry training, and an additional communications course. To date, 106 patients have had their initial visit with a community paramedic where consent was reconfirmed and initial assessment completed and documented using the electronic interfaces developed specifically for the trial. There have been no safety concerns, 100% timely oversight by the family health team and only 1 instance where a community paramedic initiated a call to 911 for emergency care.

## Authors’ information

Ian R Drennan, Katie N Dainty and Paul Hoogeveen are co-first authors.

## Electronic supplementary material

Additional file 1: **Markham Stouffville Hospital Research Ethics Board Approval.** (PDF 226 KB)

Additional file 2: **St. Michael’s Hospital Research Ethics Board Approval.** (PDF 41 KB)

Additional file 3: **Informed Consent Form.** (DOCX 23 KB)

Additional file 4: **Informed Consent Form Substitute Decision-Maker.** (DOCX 25 KB)

## Abbreviations

AIC: Akaike’s information criterion
CCAC: Community Care Access Center
COPD: chronic obstructive pulmonary disease
DM: diabetes mellitus
ED: emergency department
EMR: electronic medical records
EMS: emergency medical services
ePCR: electronic patient care records
EQ-5D 3L: Euroqol-5D 3L
EPIC: Expanding Paramedicine in the Community
HF: heart failure
ICES: Institute for Clinical Evaluative Sciences
IQR: interquartile range
ITT: intention-to-treat
KT: knowledge translation
MITT: modified intention-to-treat
MOHLTC: Ministry of Health and Long-term Care
OLS: ordinary least squares
PHIPA: Personal Health Information Privacy Act
PIPEDA: Personal Information Protection and Electronic Documents Act
QALY: quality adjusted life years
RCT: randomized controlled trial
SD: standard deviation.
