# Supplementary material for: Expanding Paramedicine in the Community (EPIC): study protocol for a randomized controlled trial
Source: Trials. 2014 Dec 2;15:473. doi: 10.1186/1745-6215-15-473 (PMC4289358; doi:10.1186/1745-6215-15-473)
Supplement: Supplementary file 3 — Additional file 3: Informed Consent Form. (DOCX 23 KB) [file 13063_2014_2337_MOESM3_ESM.docx]

**CONSENT TO PARTICIPATE IN A RESEARCH STUDY**

**Title** Expanding Paramedicine in the Community (EPIC)

**Investigator** Dr. At Corresponding Health Team
Name of Health Team

Dr. Laurie Morrison
Rescu – St. Michael’s Hospital

**Introduction**

You are being asked for your participation in the research study mentioned above being conducted at the Corresponding Health Team clinic at which you are a patient. You were selected as you have been diagnosed with Diabetes Mellitus (DM), Congestive Heart Failure (CHF) and/or Chronic Obstructive Pulmonary Disease (COPD). Before agreeing to take part in this research study, it is important that you read the following information. It includes details we think you need to know in order to decide if you wish to take part in the study. You should not provide your consent until you are sure you understand the information.

##### Background and Purpose

Initiatives aimed at reducing Emergency Department (ED) wait times and improved Community Health initiatives are major priorities in Canada. Three of the most common chronic diseases worldwide are Diabetes Mellitus (DM), Congestive Heart Failure (CHF) and Chronic Obstructive Pulmonary Disease (COPD). Paramedics are a highly trained, underutilized resource: they are mobile in the Community, are able to assess and treat acute and chronic patients, and can apply their clinical experience to potentially intervene and prevent unnecessary ED visits and hospitalizations for patients with DM, CHF, and COPD. The primary aim of this study is to determine whether such an intervention results in a decreased likelihood of hospital admission.

Study Design

Suitable patients diagnosed by their Family Physician with COPD, CHF, and/or DM from the Corresponding Health Team clinic were chosen be included in this study. After you had provided your consent to participate in this research study by contacting the Corresponding Health Team you were assigned to the intervention group. As such, you will be provided with assessment and treatment visits specific to your medical condition in your home by Community Paramedics working closely with the Primary Care Physicians from the Corresponding Health Team clinic.

##### Study Visits and Procedures

Community Paramedics will contact you to arrange assessment/treatment visits at your home. It is expected that there will be a minimum of 5 visits within the 1 year period but more visits can be scheduled as needed depending on the severity of your condition. The Community Paramedics will work with you to schedule appointments at your convenience between the hours of 8am to 6pm, 7 days per week. During your first appointment, the Community Paramedic will review this Study Consent form with you and will answer any questions you may have regarding the visits, study design, consent process, assessment/treatment procedures, etc. They will ask you to provide a signed copy of this form to verify that you understand the form and that any questions you had have been answered. The assessment/treatment procedures are the same as you would receive at the Corresponding Health Team clinic but are provided to you by the Community Paramedic in your home under the delegation of the Corresponding Health Team. Should you wish to withdraw from the study you may do so at any time. You will be given a copy of this form for your records.

### Risks Related to Being in the Study There are no harms, injuries or discomforts anticipated to occur during your participation in this study that would not otherwise occur in your regular assessment/treatment for your condition at the Corresponding Health Team.

### Benefits to Being in the Study Now that you have been assigned to the intervention group, your condition will be more closely monitored by Community Paramedics delegated by the Corresponding Health Team Physicians. You will receive assessment/treatment visits in your home to help manage your condition.

### Voluntary Participation Participation in any research study is voluntary. If you choose not to participate, this will have no effect on your medical care. If you decide to participate in this study you can change your mind without giving a reason, and you may withdraw from the study at any time without any effect on your care.

**Confidentiality**The Research Team is committed to respecting your privacy. They will make every effort to keep your study information private and confidential in accordance with all applicable privacy legislation. Providing your consent for participation will allow the study team members collect and analyze information regarding your diagnosis, treatment, use of Emergency Medical Services, and clinic / hospital visits going forwards as well as for the past 5 years. Your personal health information will be linked to Institute for Clinical Evaluative Sciences (ICES) databases. ICES is an independent, non-profit organization, whose core purpose is to conduct research that contributes to the effectiveness, quality, equity and efficiency of health care and health services in Ontario. Although your personal health information will be used to link with this data set, your personal health data will not be released and we will only report aggregate data to preserve anonymity. No information that reveals your identity will be collected or published. The results of the research will include information from many people grouped together so that no one person could be identified. You will not be named in any reports, publications or presentations that may come from this study.

Only members of the research team, those involved in your circle of care, the St. Michael’s Hospital Research Ethics Board, and the Markham Stouffville Hospital Research Ethics Board will have access to study related records (such as the consent form) for the purpose of monitoring the study. The study data will be kept securely for 10 years after the final publication of the study results. If you decide to withdraw from the study, you have the right to request withdrawal of your information. Please inform the Principal Investigator (Dr. At Corresponding Health Team or Dr. Laurie Morrison) or the Corresponding Health Team should you wish to withdraw from the study.

##### In Case You Are Harmed in the Study If you become ill, injured or harmed as a result of taking part in this study, you will receive care. The reasonable costs of such care will be covered for any injury, illness or harm that is directly a result of being in this study. In no way does providing consent waive your legal rights nor does it relieve the investigators, sponsors or involved institutions from their legal and professional responsibilities. You do not give up any of your legal rights by providing your consent.

**Expenses Associated with Participating in the Study**We do not anticipate that there will be any cost to you for participating in this study.

**Questions About the Study**If you have any further questions about the study please feel free to contact the study staff Jinbaek Kim, at 416.864.6060 x7840 or at [communityparamed@smh.ca](mailto:communityparamed@smh.ca).

This study was approved by the Markham Stouffville Hospital Research Ethics Board.  Although the Markham Stouffville Hospital Research Ethics Board approved this study, the research project does not involve the Hospital. If you have general questions about being a research participant, you may call Dr. Henry Solow, Chair, Markham Stouffville Hospital Research Ethics Board at (905)472-7000.

**Expanding Paramedicine in the Community (EPIC)**

### Consent

This study has been explained to me and any questions I had have been answered.

I know that I may leave the study at any time. I agree to take part in this study.

Print Study Participant’s Name Signature Date

(You will be given a signed copy of this consent form)

My signature means that I have explained the study to the participant named above. I have answered all questions.

Print Name of Person Obtaining Signature Date

Consent
