# Supplementary material for: Expanding Paramedicine in the Community (EPIC): study protocol for a randomized controlled trial
Source: Trials. 2014 Dec 2;15:473. doi: 10.1186/1745-6215-15-473 (PMC4289358; doi:10.1186/1745-6215-15-473)
Supplement: Supplementary file 1 — Additional file 1: Markham Stouffville Hospital Research Ethics Board Approval. (PDF 226 KB) [file 13063_2014_2337_MOESM1_ESM.pdf]

MARKHAM STOUFFVILLE HOSPITAL CORPORATION

April 23, 2013

Dr. Jane Philpott
379 Church Street, Ste. 202
Markham, ON
L6B 0T1

Dear Dr. Philpott:

Re: Expanding Paramedicine in the Community (EPIC)

The Research Ethics Board of Markham Stouffville Hospital has reviewed and approved the above study on February 12, 2013.

The quorum for approval did not include any member associated with this project.

If during the course of the research, changes in the approved submission or any new information that must be considered with respect to the study, these should be brought to the immediate attention of the Research Ethics Board.

All members of the Board wish you success in your research endeavours and look forward to continued follow-up regarding progress with this study.

Yours sincerely,

Dr. Henry Solow
Chair, Research Ethics Board

HS/jb

c.
L. Gatttoni, RESCU

381 Church St PO Box 1800
Markham Ontario L3P 7P3

905.472.7000
