# Supplementary material for: Expanding Paramedicine in the Community (EPIC): study protocol for a randomized controlled trial
Source: Trials. 2014 Dec 2;15:473. doi: 10.1186/1745-6215-15-473 (PMC4289358; doi:10.1186/1745-6215-15-473)
Supplement: Supplementary file 2 — Additional file 2: St. Michael’s Hospital Research Ethics Board Approval. (PDF 41 KB) [file 13063_2014_2337_MOESM2_ESM.pdf]

Research Ethics Office
Telephone: (416) 864-6060 Ext. 2557
Facsimile: (416) 864-6043
E-mail: pateld@smh.toronto.on.ca

St. Michael's

Inspired Care.
Inspiring Science.

April 09, 2013

Dr. Laurie Morrison,
Rescu Program Program,
St Michael's Hospital

Dear Dr. Morrison,

Re: REB# 13-086[C] - Expanding Paramedicine in the Community: EPIC

**REB APPROVAL:**	**Original Approval Date**	**April 09, 2013**
	**Annual/Interval Review Date**	**09 April, 2014**

Thank you for your application submitted on **21 March, 2013**. The above noted study has been reviewed through an expedited/delegated process (not by Full Board review). The views of the St. Michael's Hospital (SMH) Research Ethics Board (REB) have been documented and resolved.

The REB approves the study as it is found to comply with relevant research ethics guidelines, as well as the Ontario Personal Health Information Protection Act (PHIPA), 2004. The REB hereby issues approval for the above named study for a period of 12 months from the date of this letter. Continuation beyond that date will require further review of REB approval.

In addition, the following documents have been reviewed and are hereby approved:

1. Protocol (ver. 1; 20Mar2013)
   (Study activity at St. Michael's Hospital - Data linkage)

Furthermore, the following documents have been received and are acknowledged:

1. Information Letter (ver. 9Apr2013)
2. Consent Form (ver. 9Apr2013)

During the course of this investigation, any significant deviations from the approved protocol and/or unanticipated developments or significant adverse events should immediately be brought to the attention of the REB.

Please note that if a Clinical Trial Agreement is required, it must be submitted to the Office of Research Administration for review and approval. Any additional institutional approvals **must be** coordinated and approved through the Office of Research Administration prior to initiation of this research. All drug dispensing must be coordinated through the Research Pharmacy at 416-864-5413.

The St. Michael's Hospital (SMH) Research Ethics Board (REB) operates in compliance with the Tri-Council Policy Statement Ethical Conduct for Research Involving Humans, the Ontario Personal Health Information Protection Act, 2004, and ICH Good Clinical Practice Consolidated Guideline E6, Health Canada Part C Division 5 of the Food and Drug Regulations, Part 4 of the Natural Health Product Regulations, and the Medical Devices regulations. Furthermore, all investigational drug trials at SMH are conducted by Qualified Investigators (as defined in the latter document).

With best wishes

☐ Dr. Bob Hyland
Chair, Research Ethics Board

☒ Dr. Brenda McDowell
Vice Chair, Research Ethics Board

BH/BJM/aml

St. Michael's Hospital, 30 Bond Street, Toronto, ON M5B 1W8 Canada T 416.360.4000
Fully affiliated with the University of Toronto. **stmichaelshospital.com**
